# The Necrosis-Avid Small Molecule HQ4-DTPA as a Multimodal Imaging Agent for Monitoring Radiation Therapy-Induced Tumor Cell Death

**DOI:** 10.3389/fonc.2016.00221

**Published:** 2016-10-21

**Authors:** Marieke A. Stammes, Azusa Maeda, Jiachuan Bu, Deborah A. Scollard, Iris Kulbatski, Philip J. Medeiros, Riccardo Sinisi, Elena A. Dubikovskaya, Thomas J. A. Snoeks, Ermond R. van Beek, Alan B. Chan, Clemens W. G. M. Löwik, Ralph S. DaCosta

**Affiliations:** ^1^Department of Radiology, Leiden University Medical Center, Leiden, Netherlands; ^2^Percuros BV, Enschede, Netherlands; ^3^Princess Margaret Cancer Center, University Health Network, Toronto, ON, Canada; ^4^Department of Medical Biophysics, University of Toronto, Toronto, ON, Canada; ^5^STTARR Innovation Centre, University Health Network, Toronto, ON, Canada; ^6^LCBIM, Institute of Chemical Sciences and Engineering, Swiss Federal Institute of Technology of Lausanne (EPFL), Lausanne, Switzerland; ^7^Department of Radiology, Erasmus Medical Center, Rotterdam, Netherlands; ^8^Techna Institute, University Health Network, Toronto, ON, Canada

**Keywords:** radiation therapy, cell death, multimodal imaging, fluorescence imaging, cyanine, necrosis avid contrast agent, treatment response, cancer

## Abstract

**Purpose:**

Most effective antitumor therapies induce tumor cell death. Non-invasive, rapid and accurate quantitative imaging of cell death is essential for monitoring early response to antitumor therapies. To facilitate this, we previously developed a biocompatible necrosis-avid near-infrared fluorescence (NIRF) imaging probe, HQ4, which was radiolabeled with ^111^Indium-chloride (^111^In-Cl_3_) via the chelate diethylene triamine pentaacetic acid (DTPA), to enable clinical translation. The aim of the present study was to evaluate the application of HQ4-DTPA for monitoring tumor cell death induced by radiation therapy. Apart from its NIRF and radioactive properties, HQ4-DTPA was also tested as a photoacoustic imaging probe to evaluate its performance as a multimodal contrast agent for superficial and deep tissue imaging.

**Materials and methods:**

Radiation-induced tumor cell death was examined in a xenograft mouse model of human breast cancer (MCF-7). Tumors were irradiated with three fractions of 9 Gy each. HQ4-DTPA was injected intravenously after the last irradiation, NIRF and photoacoustic imaging of the tumors were performed at 12, 20, and 40 h after injection. Changes in probe accumulation in the tumors were measured *in vivo*, and *ex vivo* histological analysis of excised tumors was performed at experimental endpoints. In addition, biodistribution of radiolabeled [^111^In]DTPA-HQ4 was assessed using hybrid single-photon emission computed tomography–computed tomography (SPECT–CT) at the same time points.

**Results:**

*In vivo* NIRF imaging demonstrated a significant difference in probe accumulation between control and irradiated tumors at all time points after injection. A similar trend was observed using *in vivo* photoacoustic imaging, which was validated by *ex vivo* tissue fluorescence and photoacoustic imaging. Serial quantitative radioactivity measurements of probe biodistribution further demonstrated increased probe accumulation in irradiated tumors.

**Conclusion:**

HQ4-DTPA has high specificity for dead cells *in vivo*, potentiating its use as a contrast agent for determining the relative level of tumor cell death following radiation therapy using NIRF, photoacoustic imaging and SPECT *in vivo*. Initial preclinical results are promising and indicate the need for further evaluation in larger cohorts. If successful, such studies may help develop a new multimodal method for non-invasive and dynamic deep tissue imaging of treatment-induced cell death to quantitatively assess therapeutic response in patients.

## Introduction

The International Agency for Research on Cancer estimated that globally in 2012, 14.1 million new patients were diagnosed with cancer and that this number will increase to more than 20 million in 2025 (1). After diagnosis, most patients with solid tumors undergo surgery, radiotherapy and/or chemotherapy, and may be followed up with alternative treatments. Conventional methods for monitoring antitumor treatment response are based on anatomical imaging, e.g., X-ray, magnetic resonance imaging (MRI) and computed tomography (CT) every 6–8 weeks during the course of treatment as described in the Response Evaluation Criteria In Solid Tumors (RECIST) (2). Although RECIST provides a standardized guideline, assessment of treatment efficacy based on gross tumor size alone may be insufficient for certain organs and treatments (3). Moreover, volumetric change in tumor size based on conventional imaging may be a delayed indicator of treatment effectiveness (4), unnecessarily exposing patients to the side effects of additional ineffective treatments, and postponing treatment adjustment. Thus, there is a need for novel imaging methods to assess tumor response early and at a cellular/molecular level in order to determine treatment efficacy accurately and adjust the therapy based on tumor response (5, 6). Ideally, such methods would be non-invasive, clinically practical, and have sufficient sensitivity and specificity for tumor cell death in real time.

Imaging of treatment-induced tumor necrosis may facilitate quantitation of early treatment response in solid tumors as an alternative to the conventional radiological volumetric imaging. First, antitumor therapies, such as radiation therapy are known to induce several forms of tumor cell death that will often lead to secondary necrosis (7–9). Second, necrosis is primarily induced by external factors that cause physiochemical damage compared to apoptosis that can occur in any tissues during normal development and cell turnover (10, 11), making necrosis-based imaging method suitable to distinguish cell death induced by antitumor therapies. Finally, tumor necrosis, secondary to ischemia and insufficient vascularization to support a rapidly proliferating tumor mass (12), has been positively correlated with the aggressiveness of cancer, and, therefore, has been used as a diagnostic biomarker for cancer staging (13–18). Thus, exogenous imaging contrast agents that specifically bind to necrotic tumor cells *in vivo* could enable accurate determination of treatment effects and disease staging, as well as earlier prediction of treatment outcomes for solid tumors (19).

Accurate quantification of tissue necrosis may have wide clinical relevance compared to conventional practice, especially in monitoring the efficacy of antitumor therapies at earlier stages. Existing necrosis-based imaging agents can be divided into two general groups: (1) MRI and CT contrast agents that enhance endogenous tissue necrosis contrast non-specifically by enabling visualization of the presence of an avascular necrotic core and (2) positron emission tomography (PET) and single-photon emission computed tomography (SPECT) contrast agents that are specifically targeted to endogenous necrotic tissue (8, 13–18, 20–29). Non-specific tissue necrosis-imaging agents will likely fall into abeyance when affordable necrosis-specific agents become clinically available. Thus far, only a few agents have been considered clinically applicable, including necrosis-avid photosensitizer hypericin (Oncocidia™) (30–32) and ^131^Iodine-conjugated Tumor Necrosis Targeting monoclonal antibody (TNT-3, Peregrine Pharmaceuticals, CA, USA) (24, 33). While clinical feasibility has been shown for both agents (34), several drawbacks may hinder their wide-spread clinical adoption (27, 30, 35–37). For example, hypericin is phototoxic, poorly soluble, and aggregates rapidly. Monoclonal antibodies are relatively large in size, have long circulation times, may induce host immune response, and are expensive to develop using good manufacturing practices (GMP) (35–38).

Recognizing the biological significance of tumor necrosis as a hallmark of tumor response to treatment and the need for alternative imaging methods to measure treatment-induced solid tumor necrosis, we previously developed a biocompatible near-infrared fluorescence (NIRF), water-soluble imaging probe called HQ4. HQ4 is economical to produce, is non-phototoxic, and binds specifically to cells with compromised cell membrane integrity (38). We validated HQ4-diethylene triamine pentaacetic acid (HQ4-DTPA) as a necrosis-avid contrast agent histologically by demonstrating localization of HQ4-DTPA in necrotic tumors, and indicated that HQ4-DTPA could be made more clinically practical by addition of a radioactive moiety (38). Building on these results, in the current study, we investigated the utility of HQ4-DTPA as a necrosis-imaging agent *in vivo* to measure tumor response to radiation therapy. Since radiotherapy is used to treat over 50% of cancer patients (39), the translational value of HQ4-DTPA is promising.

In this study, we examined a relatively high-dose-per-fraction treatment scheme (3 × 9 Gy) to induce tumor cell death. This regime was calculated based on a biological equivalent dose (BED) that is clinically relevant to 60 Gy for 2 Gy fractions, given a known α/β ratio of MCF-7 cells, comparable to the average α/β ratio of humans, and the incomplete repair model. We investigated a trimodal HQ4-DTPA imaging (photoacoustic, NIRF, SPECT) approach to measure tumor response to radiation therapy in a MCF-7 human breast cancer mouse xenograft model. We reasoned that the addition of photoacoustic imaging would overcome some of the disadvantages associated with SPECT and NIRF, such as the exposure to ionizing radiation emitted from radionuclides and the limited penetration depth (40), respectively. Photoacoustic imaging may also be ideal for routine clinical use as it is easily accessible, minimally invasive, and technologically inexpensive compared to conventional imaging methods (CT, MRI). The results of this work demonstrate the feasibility of using the multimodal (NIRF, photoacoustic, SPECT) HQ4-DTPA probe *in vivo* for longitudinal measurement of solid tumor necrosis in response to clinically relevant high-dose radiotherapy.

## Materials and Methods

### HQ Preparation

HQ4-DTPA was obtained from Ilumicare BV (Rotterdam, The Netherlands). HQ4-DTPA was synthesized as previously described (38). For phantom studies, dilutions of HQ4-DTPA were prepared in phosphate buffered saline (PBS) at various concentrations (12.5, 25, 50, and 100 μM). For *in vivo* mouse studies, 100 μL that represents 10 nmol HQ4-DTPA was injected via the tail vein. To label HQ4-DTPA with ^111^InCl_3_, HQ4-DTPA was dissolved in 0.1 M HEPES (10 μg/100 μL) (41) and incubated with ^111^InCl_3_ (35 MBq; Nordion, Vancouver, BC, USA). After 30 min of incubation on a shaker, labeling was validated with instant thin layer chromatography (ITLC). In all cases, labeling efficacy was greater than 90%.

### Cell Culture

GFP-fluorescent MCF-7 human breast cancer cells (kindly provided by Dr. Shirley Wu, Leslie Dan Faculty of Pharmacy, University of Toronto) were grown in DMEM Medium supplemented with 10% fetal bovine serum and 1% Pen-Strep in a humidified incubator at 37°C and 5% CO_2_. Cells were trypsinized, counted, and suspended in 10% PBS before further use.

### Animal Studies

**Figure 1:**
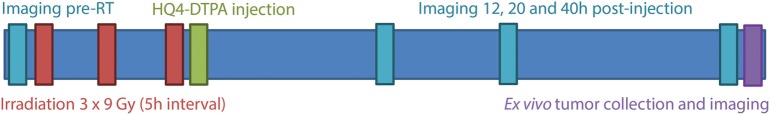
**Experimental schedule**. Three fractions of 9 Gy irradiation were delivered with a 5-h interval and HQ4-DTPA was injected at the end of the irradiation schedule. *In vivo* photoacoustic and fluorescence imaging were performed at 12, 20, and 40 h following the injection.

All animal procedures were conducted in accordance with appropriate regulatory standards under protocols AUP#2407 and #3004 approved by the University Health Network Institutional Animal Care Committee, and conform to the institutional guidelines for the proper and humane use of animals in research. Eight to 10-week-old female athymic nude mice (NCRNU-F strain) were obtained from Taconic Biosciences (Hudson, NY, USA). Approximately 2 × 10^6^ MCF-7-GFP cells were injected subcutaneously in both sides of the mouse scapularis region and were allowed to grow for 3–4 weeks until they reached approximately 5 mm in diameter, as measured using a caliper. All experimental procedures were conducted under isoflurane gas anesthesia (2–3%, 0.8 L/min). All animal experiments were performed following the treatment schedule shown in Figure 1. Briefly, pre-treatment images were obtained prior to irradiation to determine the size of the tumors based on bulk tumor GFP fluorescence. Tumor GFP fluorescence intensity is a delayed indicator of tumor response to irradiation since the GFP protein has a half-life of ~26 h (42), ergo, GFP fluorescence intensity was not used to quantify tumor response following irradiation.

### Radiation Treatment and HQ4-DTPA Administration

All irradiation procedures were performed using a small animal irradiation system (XRad 225Cx, Precision X-Ray Inc., North Branford, CT, USA) at a photon energy of 225 kVp and a tube current of 13 mA. Tumors were localized using x-ray fluoroscopy prior to irradiation. A 1.5 cm circular collimator was used to irradiate tumors at a dose rate of 2.9 Gy/min. The dose rate was measured using radiochromic films and a solid water phantom, as described previously (43). After delivery of the last radiation fraction, HQ4-DTPA was injected via the tail vein and anesthetized mice were imaged with each modality at 12, 20, and 40 h following injection (Figure 1).

### Fluorescence Imaging

*In vivo* and *ex vivo* fluorescence images of GFP and HQ4-DTPA signals in MCF-7 tumors were obtained using the IVIS Spectrum imaging system (Perkin Elmer Inc., Waltham, MA, USA). GFP fluorescence signal was collected with an excitation wavelength of 465 nm and an emission wavelength of 500 nm (±20 nm). HQ4-DTPA NIRF signal was collected with an excitation wavelength of 675 nm and an emission wavelength of 720 nm (±20 nm).

### Photoacoustic Imaging

Tissue phantom, *in vivo* and *ex vivo* photoacoustic imaging of MCF-7 tumors was performed using the Vevo LAZR system (FujiFilm VisualSonics Inc., Toronto, ON, Canada) with a 21 MHz center-frequency transducer. To prepare the phantom, HQ4-DTPA samples prepared at different concentrations (12.5, 25, 50, and 100 μM) were passed through polyethylene tubes that were placed on a piece of sliced turkey breast, having an approximate thickness of 2.5 mm. Additional layers of meat were added to simulate various thicknesses of tissues. Photoacoustic images were obtained after the addition of each layer. For all experiments, 3D photoacoustic and ultrasound images were acquired simultaneously with a single wavelength of 700 nm for HQ4-DTPA, and the built-in Spectro mode was used to obtain the absorption spectrum from 680 to 900 nm.

### SPECT-CT Imaging

Mice were imaged at 12, 20, and 40 h after intravenous injection of [^111^In]DTPA-HQ4. Mice were anesthetized by inhalation of 2% isoflurane in medical grade air. Imaging was performed on a nanoSPECT/CT system (Bioscan Inc., Washington, DC, USA) with four NaI(Tl) detectors fitted with 1.4-mm multi-pinhole collimators (resolution <1.2 mm at full-width-half-maximum). Cone beam CT images were acquired first (180 projections, 45 kVp), followed by the SPECT images. Photons were accepted from the 10% window centered on both the 245 keV and 171 keV photopeaks of ^111^In. A total of 24 projections were obtained in a 256 × 256 matrix for a total of 45 min. The CT slices were reconstructed using a filtered back-projection algorithm, whereas the SPECT slices were reconstructed using an ordered subset expectation maximization (OSEM) algorithm with four subsets and nine iterations. CT and SPECT images were anatomically co-registered using the InVivoScope software (Bioscan/inviCRO, Boston, MA, USA).

Three mice were sacrificed after each experimental time point. Tissues were excised, weighed, and counted for radioactivity (PerkinElmer Wallac 1480 Wizard 3″ gamma-counter, Waltham, MA, USA) along with a standard of the injected dose, so that the decay-corrected uptakes of HQ4-DTPA were determined as the percentage of the injected dose per gram (% ID/g). The % ID/g was calculated as follows: [(MBq measured in tissue/injected dose) × 100%/weight of tissue]. The total injected dose per mouse was equal to the difference between the pre- and post-injection syringe radioactivity, as measured by a CRC-15R dose calibrator (Capintec, Ramsey, NJ, USA).

### *Ex Vivo* Fluorescence Imaging and Autoradiography

Tumors were resected at the experimental endpoints, and were either embedded in OCT compound and snap frozen in liquid nitrogen, or fixed in formalin. Frozen sections were imaged using a phosphor imager (Cyclone Plus, Perkin Elmer) to detect ^111^In radioactivity. The same sections were subsequently imaged to measure HQ4-DTPA fluorescence with an excitation wavelength of 650 nm using TissueScope system (Huron Technologies). Formalin-fixed tissue sections were subjected to Hemotoxylin and Eosin (H&E) staining and TdT-mediated dUTP Nick-End Labeling (TUNEL) staining (Promega, Madison, WI, USA) to detect radiation-induced tumor cell death including necrosis (44, 45).

### Statistical Analysis

All statistical analyses were performed using GraphPad Prism^®^ software (GraphPad Software, San Diego, CA, USA). Student’s *t*-test was used to compare two sets of data, and two-way repeated measures ANOVA with Bonferroni post-test was used for serial imaging data. *p* < 0.05 was considered significant, and error bars represent the mean ± SEM.

## Results

### HQ4-DTPA as a Photoacoustic Contrast Agent

**Figure 2:**
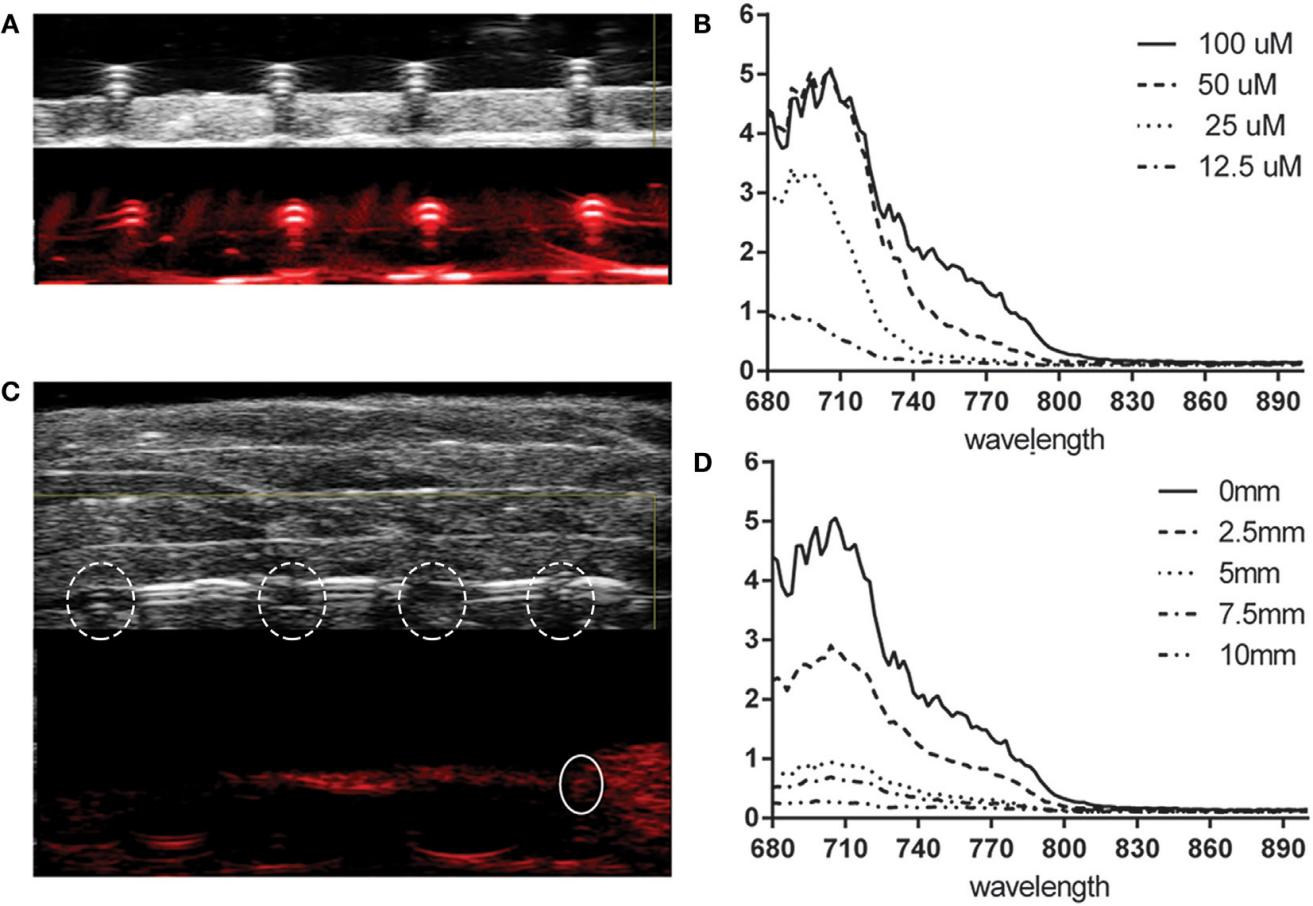
**Photoacoustic property of HQ4-DTPA**. **(A)** Representative ultrasound (top) and photoacoustic (bottom) images of HQ4-DTPA in a tube phantom at different concentrations (from left to right: 12.5, 25, 50, and 100 μM). The photoacoustic image was acquired at 700 nm. **(B)** Corresponding absorption spectra of HQ4-DTPA for the different concentrations. **(C)** Representative ultrasound (top) and photoacoustic (bottom) images of HQ4-DTPA in the same tube phantom as in **(A)**, covered with 10-mm-thick meat. The dashed circles indicate the location of tubes, and the solid circle indicates the photoacoustic absorption of the tube containing 100 μM HQ4-DTPA. **(D)** The corresponding absorption spectra of 100 μM HQ4-DTPA with various thicknesses of meat covering the tube.

To evaluate the application of HQ4-DTPA in addition to the NIRF property that was described previously (38), the photoacoustic property of carboxylated cyanine HQ4-DTPA was tested in a phantom composed of transparent plastic tubes. As seen in Figures 2A,B, HQ4-DTPA absorption increased with increasing concentration, demonstrating a peak at around 710 nm excitation. The photoacoustic absorption spectrum was similar to its known fluorescence spectrum (38), supporting its use as an extrinsic photoacoustic contrast agent.

To further characterize its performance as a photoacoustic contrast agent, multiple layers of meat were added over top of the tube phantoms to simulate a tissue thickness of up to 1 cm. After the addition of each layer of meat, fluorescence and photoacoustic images, as well as photoacoustic absorption spectra, were acquired. In this way, we represented similar scattering and absorption patterns to those found in the human body. The fluorescence signals derived from the different concentrations were indistinguishable by the addition of the first layer of turkey breast tissue (2.5-mm thick) (data not shown). The photoacoustic intensity of the agent in the tubes was, however, detectable with layers up to 10 mm in total thickness at the highest concentration of HQ4-DTPA (100 μM) (Figure 2C). Figure 2D shows the PA absorption spectra of 100 μM HQ4-DTPA with the addition of 2.5-mm-thick tissue layers.

### *In Vivo* Serial Photoacoustic and Fluorescence Imaging of HQ4-DTPA Accumulation in Irradiated Tumors

**Figure 3:**
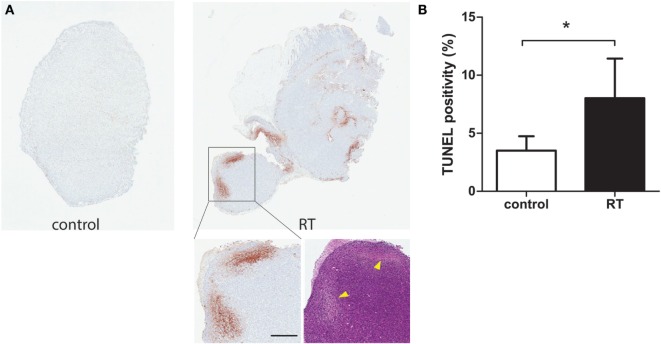
**Histological analysis of cell death after irradiation**. **(A)** Representative images of TUNEL and H&E staining for control and irradiated tumor resected 40 h after irradiation. The arrowheads point to necrotic areas. Scale bar = 500 μm. **(B)** Quantified TUNEL positivity for tumors resected 40 h after irradiation, expressed as % positivity (*n* = 7/group, *p < 0.05).

**Figure 4:**
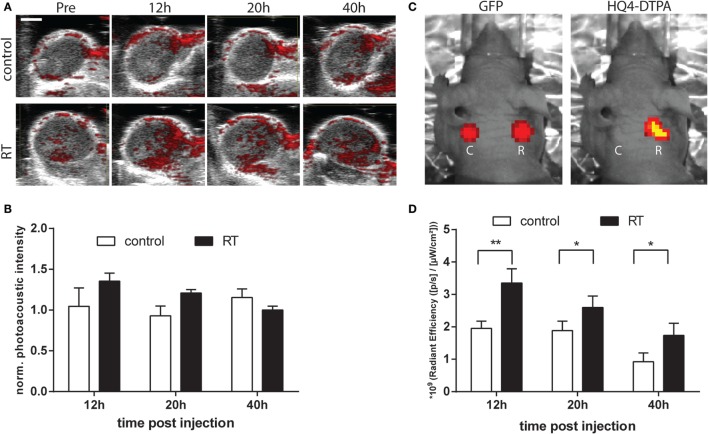
***In vivo* photoacoustic and fluorescence imaging of tumors**. **(A)** Representative photoacoustic images of control and irradiated tumors prior to and at 12, 20, and 40 h following injection of HQ4-DTPA. The photoacoustic image was acquired at 700 nm and an accumulation of HQ4-DTPA inside the irradiated tumor was observed. Scale bar = 2 mm. **(B)** Measured photoacoustic intensity at different time points (*n* = 3/group). **(C)** Representative fluorescence images of the GFP-MCF7 tumor and HQ4-DTPA in control (C) and irradiated (R) tumors. **(D)** Measured fluorescence intensity of HQ4-DTPA at different time points (*n* = 10/group, *p < 0.05, **p < 0.01).

TdT-mediated dUTP nick-end labeling staining of irradiated tumor demonstrated over a two-fold difference in tumor cell death in tumors irradiated with three fractions of 9 Gy (27 Gy total), compared to non-irradiated control tumors (Figures 3A,B). H&E staining was performed to confirm the TUNEL positive area as necrotic. The arrowheads in the image mark the difference in H&E staining between healthy and necrotic tissue. Based on those results, the same irradiation treatment regimen was used for all subsequent experiments. As demonstrated in Figure 4A, the photoacoustic images demonstrated accumulation of HQ4-DTPA inside the treated tumor mass, while some endogenous photoacoustic signals were observed in the outer rim of the tumor in both control and irradiated tumors. Figure 4B demonstrated a trend for increased accumulation of HQ4-DTPA in irradiated tumors compared to control tumors, although the difference was not statistically significant. The fluorescence images (Figures 4C,D) showed an approximate 1.8-fold increase in HQ4-DTPA accumulation in the irradiated subcutaneous tumors compared to non-irradiated controls, most notably at 12 h post-radiotherapy.

### Validation of Photoacoustic and Fluorescence Imaging of HQ4-DTPA in *Ex Vivo* Tissues

**Figure 5:**
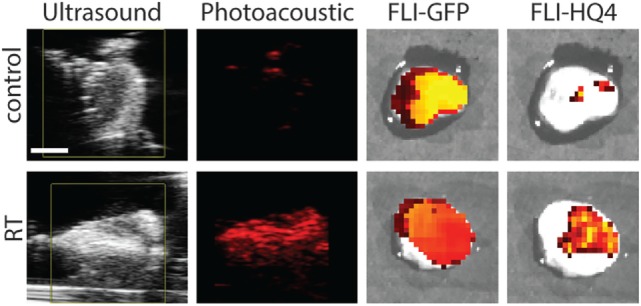
***Ex vivo* photoacoustic and fluorescence imaging**. Representative ultrasound, photoacoustic, GFP fluorescence (FLI-GFP) and HQ4-DTPA fluorescence (FLI-HQ4) images of control and irradiated tumors resected 40 h after injection of HQ4. Scale bar = 2 mm.

To validate *in vivo* observations, control and irradiated tumors were resected 40 h following injection of HQ4-DTPA and subsequently imaged by photoacoustic and fluorescence systems. The resected masses were confirmed to be tumors based on the GFP fluorescence signal. As seen in Figure 5, increased HQ4-DTPA accumulation in an irradiated tumor was observed based on photoacoustic and fluorescence images. This indicated that the increased accumulation of HQ4-DTPA was specific to radiation-induced tumor cell necrosis in tumors. Since the skin covering the xenografted tumor was removed during resection, there was less interference from the intrinsic hemoglobin signal from blood vessels in the skin.

### *In Vivo* Biodistribution and *Ex Vivo* Validation of ^111^In Radiolabeled HQ4-DTPA

**Figure 6:**
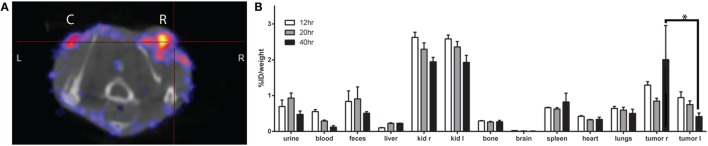
**SPECT-CT of [^111^In]DTPA-HQ4-DTPA biodistribution**. **(A)** Representative transversal SPECT–CT image of the mouse with control (C) and irradiated (R) tumor 40 h following injection. **(B)** Measurement of [^111^In]DTPA-HQ4 biodistribution in% ID/weight in different organs at various time points (12, 20, and 40 h post injection), where irradiated tumor (“tumor *r*”) shows higher accumulation of the probe compared to control (“tumor *l*”) (*n* = 3, *p < 0.05).

**Figure 7:**
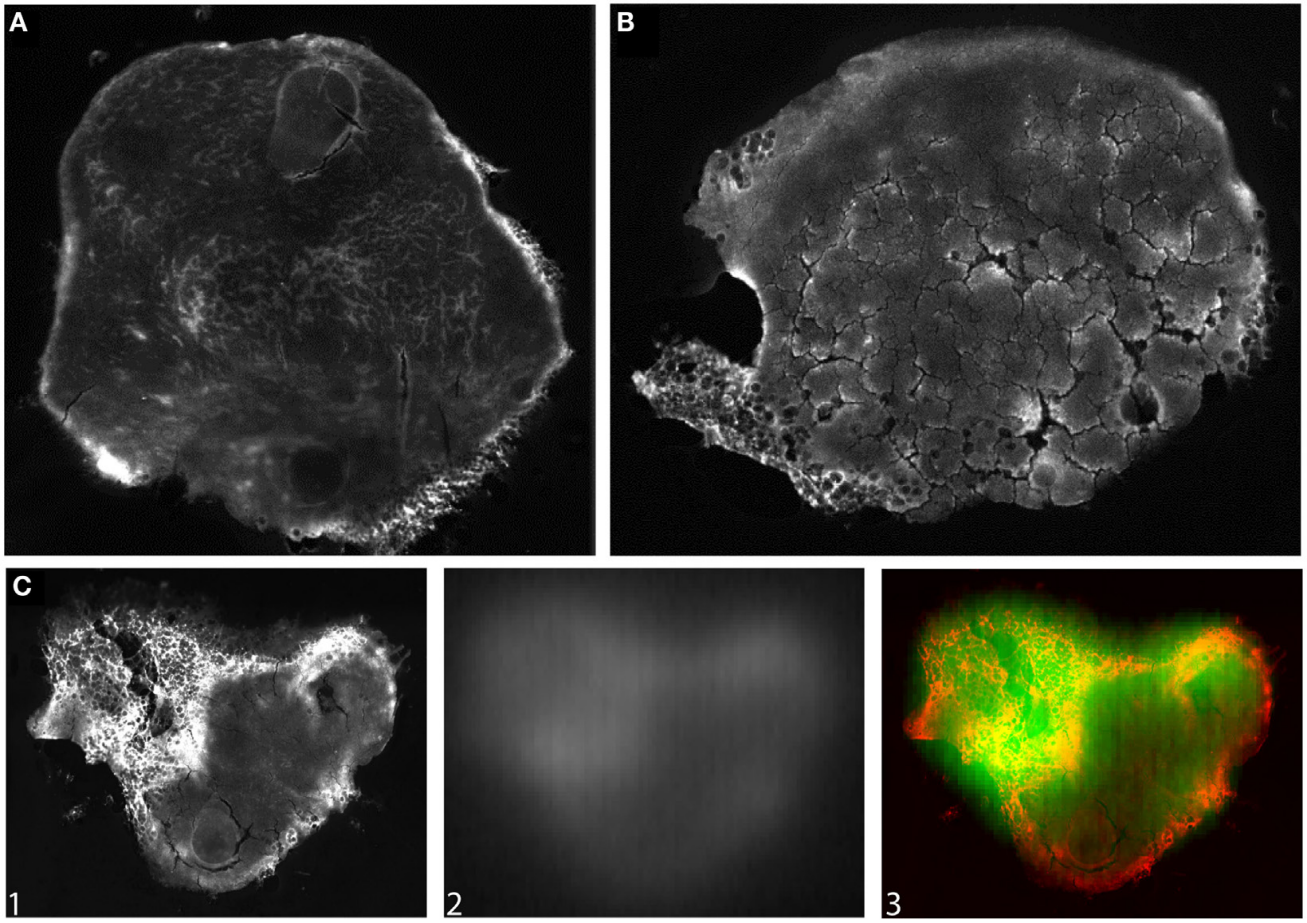
***Ex vivo* fluorescence and autoradiography images of tumors**. Representative images of **(A)** control and **(B)** irradiated tumor resected 40 h following injection of [^111^In]DTPA-HQ4. The tumors were imaged for HQ4-DTPA fluorescence. **(C)** Overlay (C3) of HQ4-DTPA fluorescence (C1-red) and ^111^InCl_3_ radioactivity (C2-green) in a tumor, illustrating co-localization of the two signals.

SPECT-CT was performed to quantify whole-body biodistribution of [^111^In]DTPA-HQ4 in MCF-7 tumor-bearing mice following the experimental treatment schedule shown in Figure 1. Radiolabelling efficiency of HQ4-DTPA was determined to be greater than 90%. Significantly higher accumulation of [^111^In]DTPA-HQ4 in irradiated tumors was observed compared to controls in the same mice 40 h after probe injection [tumor-to-background ratio (TBR) = 1.8, *p*_40 h_ = 0.03] (Figure 6A), thus confirming HQ4’s specificity for necrotic tissues and suggesting the kinetics of HQ4-DTPA accumulation. Measurements of radioactivity in various resected organs demonstrated that [^111^In]DTPA-HQ4 was concentrated in the excreting organs with a peak in the kidneys, suggesting that the renal system was the main excreting route (Figure 6B). Lastly, the tumors were resected and imaged for [^111^In]DTPA-HQ4 using autoradiography and fluorescence. The autoradiographic images revealed a clear difference in structural characteristics between the irradiated and control tumors (Figures 7A,B). The internal tissue organization of the non-irradiated tumor was cohesive and showed a clear cellular pattern with a homogeneous color. By contrast, the irradiated tumor showed a high level of disorganization. Furthermore, the overlay (C3) of fluorescence (C1-red) and autoradiography (C2-green) images showed a high degree of co-localization of ^111^In-Cl_3_ and HQ4-DTPA fluorescence signal (Figure 7C).

## Discussion

In the current study, we evaluated HQ4-DTPA as a multimodal necrosis-avid imaging agent to assess tumor response to a clinically relevant radiotherapy dose using a MCF-7 human breast cancer mouse xenograft model. The necrosis-avid property of HQ4-DTPA for detection of chemotherapy-induced tumor cell necrosis was previously demonstrated using NIRF and SPECT (38). To extend the applicability of HQ4-DTPA to another imaging modality, we first assessed its photoacoustic property and demonstrated its distinct optical absorption peak at ~700 nm. Based on this result, multimodal imaging was performed to quantitatively evaluate the *in vivo* use of HQ4-DTPA to detect tumor response to radiotherapy using a fractionated irradiation scheme (3 × 9 Gy). Our *in vivo* fluorescence results demonstrated an increase in HQ4-DTPA signal in irradiated tumors compared to non-irradiated tumors *in vivo* for up to 40 h after treatment, indicating specific and sustained accumulation of HQ4-DTPA in irradiated tumors. These data were supported by *ex vivo* NIRF and photoacoustic imaging of control and irradiated tumors. Lastly, we used SPECT-CT to quantitate the biodistribution of HQ4-DTPA, demonstrating HQ4-DTPA accumulation in irradiated tumors and clearance of unbound HQ4-DTPA mostly via kidneys, which was visualized at all time points. Collectively, our data indicated that HQ4-DTPA may be used as a multimodal necrosis-specific imaging agent. The data also suggested that HQ4-DTPA may be used clinically in the future to monitor solid tumor response to radiation therapy in a practical time frame.

Radiation therapy was selected as the treatment modality for the breast cancer model in the present study because of its wide-spread clinical usage. Breast conserving surgery is the standard treatment for localized breast cancer in combination with (neo-)adjuvant therapies (46), such as radiation therapy, which has been shown to reduce local recurrence (47). Radiation therapy is commonly administered in a conventional fractionated schedule (25 fractions of 2 Gy) on the breast with an additional boost of up to 10 Gy on the lumpectomy cavity (47). Since such treatment schemes cannot be easily replicated in a relevant manner in animal models, we selected a radiation regimen that is isoeffective to a clinically relevant fractionated irradiation regimen based on a BED of 60 Gy in 2 Gy fractions (48, 49). Thus, the rationale for three fractions of 9 Gy with a 5-h interval was based on the BED for 60 Gy, calculated using the α/β ratio of MCF-7 cells (4.62) (50, 51) and by taking into account the incomplete repair model based on the halftime of recovery from radiation damage in murine skin. Although the radiation regimen used in our study may not be used routinely or be clinically practical, we assumed that the regimen was appropriate to mimic the total radiation dose given in cancer patients as the BED is used to compare the relative effectiveness of different radiation protocols that vary in fraction size.

The NIRF properties of carboxylated cyanine HQ4-DTPA and its radioactive labeled variant [^111^In]DTPA-HQ4 have been previously demonstrated by our group (38). Although nuclear imaging overcomes the limited tissue penetration depth of NIRF imaging (40, 52), it has its drawbacks, including radiation safety, cost of radioactive materials, limited temporal sensitivity, and the lack of anatomical detail (53). Photoacoustic imaging overcomes such limitations and offers a novel and clinically relevant means of imaging HQ4-DTPA *in vivo*. Since photoacoustic imaging includes ultrasound imaging, both anatomical and functional information can be obtained simultaneously in real time. Photoacoustic imaging can image beyond the depth limitation of fluorescence imaging to more than 5 cm (54), making it suitable for imaging deeper tumors. In the current *in vitro* experiments, we distinguished a specific photoacoustic signal at a maximum depth of 1 cm, achieving five times the tissue depth of NIRF imaging. However, depth imaging beyond 1 cm could not be performed due to the inherent property of the high-frequency ultrasound transducer (21 MHz) used in our study. Photoacoustic imaging depth may be increased by using a lower frequency transducer, but at the expense of reduced detection sensitivity (55). Alternatively, a higher concentration of the probe may facilitate detection in deeper tissue.

Despite the advantages of photoacoustic imaging, there are some technical limitations. First, photoacoustic imaging may not be a suitable method for certain organs, such as lung and brain, where acoustic impedance is different between tissue interfaces (56). However, several preclinical studies demonstrated the use of photoacoustic imaging in these organs, suggesting future use of photoacoustic imaging in a variety of organs (57–59). Second, the clinical use of photoacoustic contrast must be approached cautiously, since photoacoustic imaging visualizes any tissue-based optical absorber at a given wavelength. As such, this method detects the presence of endogenous hemoglobin, a primary optical absorber in tissues, across a broad spectral range that includes 700–750 nm, corresponding to the peak absorption of HQ4. Our photoacoustic imaging results indicated the presence of an endogenous optical absorber mostly in the periphery of control and irradiated tumors, suggesting the presence of vasculature around the tumor. The endogenous absorption limited our ability to detect HQ4-DTPA in a highly specific manner. To distinguish absorption by any contrast agent from that of endogenous absorbers, photoacoustic spectral unmixing techniques can be performed to obtain a clear overview of the contrast agent signal based on its known spectrum (60). Such techniques can be applied in future studies to visualize the accumulation of HQ4-DTPA inside the irradiated tumors in a specific manner. In addition, the imaging probe may accumulate inside tumors due to intrinsic tumor necrosis resulting in the presence of background signal in both fluorescence and photoacoustic imaging. In such cases, baseline imaging needs to be performed with the injection of HQ4-DTPA prior to initiation of an anticancer treatment.

Although NIRF imaging is widely clinically applicable, its use as a singular imaging modality to assess biological activities may be suboptimal. For example, fluorescence properties of exogenous dyes used *in vivo* are strongly influenced by the tissue microenvironment, such as hydrophobicity and pH, as well as by interactions with various proteins (61, 62). Such interactions will influence HQ4-DTPA fluorescence intensity differently in an *in vivo* environment of living cells, which may hamper quantification of probe accumulation inside necrotic tumors. These same interactions may have contributed to the differences in the time point of highest signal accumulation observed using the different imaging methods in our model, although it was not explicitly addressed in this study. To achieve absolute quantification of a probe, gamma spectroscopy or mass spectrometry should be considered (61, 62). In our study, quantification of HQ4-DTPA was achieved by measurement of radioactivity in various organs, supporting the *in vivo* imaging data in a quantitative manner.

Irradiation causes direct DNA damage and the production of reactive oxygen species (ROS), both leading to cell death. The amount and the type of cell death depend on the tumor type and the irradiation dose per fraction. For MCF-7 cells, the α/β ratio, a model of radiation effect, is relatively low compared to the higher α/β ratios for other tumors, such as Tara-1/2 (teratoma), DU145 (bladder), TSU, and UNCap (prostate) (e.g., 7–20 Gy) (51), suggesting that the treatment response may be delayed in MCF-7 tumor-bearing animals. This delayed response can be seen by our *in vivo* radioactivity-based biodistribution results demonstrating significant differences of HQ4-DTPA accumulation between the treated and control tumor 40 h after irradiation. This discrepancy may increase even more over time, requiring re-injection of the probe at a later time point or at multiple time points following radiotherapy. In addition, radiation-induced damage may be more severe when high dose of radiation is used per fraction, leading to direct tumor cell destruction as well as secondary tumor cell death (63, 64). Therefore, future studies may focus on multi-fractionated scheme with a lower fraction dose to assess whether the proposed necrosis-imaging technique is still applicable. In testing multi-fractionation schemes, the imaging technique could be initially tested in the same way so immediately after the end of the complete treatment, and later on even during the treatment process to assess its utility in adapting therapeutic regimen. In the current study, we chose to inject HQ4-DTPA immediately after the final tumor irradiation to detect early treatment response since the goal of this study was to investigate HQ4-DTPA imaging as an early indicator of radiation-induced necrosis, Collectively, future studies are warranted with multi-fractionation scheme and/or injections at multiple time points to evaluate its utility in treatment monitoring and adaptive treatment.

Overall, we have demonstrated that HQ4-DTPA can be used to objectively assess tumor response to radiation therapy. HQ4-DTPA is distinct from current clinically available necrosis-avid agents given its unique *in vivo* specificity and multimodal imaging capability. The added benefit of multimodal imaging potentially broadens its applicability in a variety of clinical settings, where tissue necrosis serves as a surrogate marker of diseases as well as response to necrosis-inducing treatments. The advantages of the small molecule [^111^In]DTPA-HQ4 include high water solubility, the photoacoustic property that enables deep tissue penetration into tissues, lack of phototoxicity, and low production costs. Unlike fluorescence imaging and SPECT, photoacoustic imaging combines the anatomical and functional properties of tissue in a 3D image. Therefore, the necrosis avid radiotracer [^111^In]DTPA-HQ4 has the potential to be clinically translated for diagnostic and prognostic purposes, as well as to predict early treatment outcome of antitumor treatments, such as radiation therapy. Additional preclinical and clinical studies are required to demonstrate the advantages of this novel imaging approach to assess early treatment efficacy and inform adaptive therapy decisions for individual patients.

## Author Contributions

The author contribution can be divided into several parts in which several authors played an essential role. Those parts and the contributions are described below: Conception of the work: MS, AM, DS, PM, ED, AC, CL, and RD. Design of the work: MS, AM, JB, DS, RS, TS, and ER. Acquisition of the work: MS, AM, JB, DS, and RS. Analysis/interpretation of the work: MS, AM, PM, TS, ER, AC, CL, and RD. Drafting the work: MS, AM, IK, and RD. Revising the work: MS, AM, DS, IK, ED, CL, and RD. Final approval: MS, AM, JB, DS, IK, PM, RS, ED, TS, EB, AC, CL, and RD. Agreement to be accountable: MS, AM, JB, DS, IK, PM, RS, ED, TS, EB, AC, CL, and RD.

## Conflict of Interest Statement

HQ™ compounds are a trade mark of Ilumicare BV, Rotterdam. The authors have no other conflicts of interest to declare.
